# Microvascular Density Analysis of Patients with Trigeminal Herpes Zoster—An Optical Coherence Tomography Angiography Study

**DOI:** 10.3390/biomedicines13071630

**Published:** 2025-07-03

**Authors:** Eliane Luisa Esser, Steven Brozmann, Sebastian Dierse, Martin Dominik Leclaire, Nicole Eter, Nataša Mihailovic, Jan Ehrchen

**Affiliations:** 1 Department of Ophthalmology, University of Muenster Medical Center, 48149 Muenster, Germany; 2 Augen-Zentrum-Nordwest, 48683 Ahaus, Germany; 3 Department of Ophthalmology, Klinikum Bielefeld Gem. GmbH, 33604 Bielefeld, Germany; 4 Department of Dermatology, University Hospital of Muenster, 48149 Muenster, Germany

**Keywords:** herpes zoster (HZ), vessel density, choriocapillaris, OCTA, varicella zoster virus (VZV), retinal thickness

## Abstract

**Purpose:** Varicella zoster virus (VZV) vasculopathy can occur in patients with herpes zoster (HZ). Our aim was to evaluate the retinal microvascular vessel density (VD) in patients with trigeminal HZ measured by optical coherence tomography angiography (OCTA). **Methods**: 48 eyes of 24 patients with HZ and 48 eyes of 24 healthy age- and gender-matched controls were included in this study. All participants underwent an OCTA examination using RTVue XR Avanti with AngioVue. The VD data of the macular 3 × 3 mm OCT angiogram of the superficial capillary plexus (SCP), the deep capillary plexus (DCP), and the choriocapillaris (CC) as well as the VD data of the optic nerve head (ONH) were extracted and analyzed. **Results:** The VD in the SCP, DCP, and CC of patients with HZ was significantly lower compared with healthy controls (*p* < 0.05). Equally, there was a noticeable reduction in the inside disk area of the ONH. There was no statistically noticeable reduction in the FAZ area and central retinal thickness. **Conclusions**: In this study, HZ patients demonstrated a decrease in the retinal VD of the SCP, DCP, ONH, and the CC. Quantitative analysis of retinal perfusion using OCTA may therefore help in the diagnosis and monitoring of HZ. Further studies must show to what extent this may be an indication of VZV-related vasculopathy and whether OCTA data can be used as a biomarker in these patients in the future.

## 1. Introduction

Herpes zoster (HZ), or shingles, is caused by the reactivation of the varicella-zoster virus (VZV), which remains latent in nerve ganglia after primary infection with chickenpox [1]. HZ has a global distribution with an age-dependent incidence, ranging from 1.2 to 3.4 per 1000 persons annually in younger adults to 3.9–11.8 per 1000 in those aged 65 years and older [2,3]. Risk factors include advancing age, immunosuppression, stress, diabetes, and comorbidities like megaloblastic anemia [4,5].

HZ is characterized by a painful vesicular rash that follows a dermatomal distribution [6]. The disease can lead to severe complications, including postherpetic neuralgia (PHN), vision loss, and, less frequently, hearing impairment [7,8,9]. However, its impact extends beyond the skin, particularly when it involves the central nervous system and its cranial nerves [10]. HZ involving the ophthalmic branch of the trigeminal nerve, known as herpes zoster ophthalmicus (HZO), presents unique challenges [9,11].

Emerging evidence suggests that HZ may also have vascular implications, potentially triggering inflammation in blood vessels and leading to disruptions in microcirculation [12,13,14]. Vasculopathies caused by VZV are believed to result from the trans-axonal spread of VZV to adjacent blood vessels, the viral infection of cerebral blood vessels, vascular remodeling, and thrombosis [15]. Vascular disturbances in this so-called VZV vasculopathy may contribute to long-term complications such as ischemic tissue damage or an increased risk of cerebrovascular events [13,16]. In fact, the risk of stroke is increased by 30% within one year after zoster [17,18]. Zoster in the area of the first branch of the trigeminal nerve is associated with a 4.3-fold risk of stroke [17].

Optical coherence tomography angiography (OCTA) is a non-invasive imaging modality that enables the detailed visualization and quantitative analysis of the retinal and choroidal microvasculature without the need for an intravenous dye injection. Its high reproducibility and ease of use have made it an increasingly valuable tool in both clinical and research settings. One of the primary quantitative metrics derived from OCTA is vessel density (VD), which reflects the proportion of the scanned area occupied by blood vessels and serves as an indicator of microvascular perfusion.

OCTA has been widely applied to evaluate retinal perfusion in systemic inflammatory diseases such as rheumatoid arthritis and systemic sclerosis, where significant alterations in the retinal microvasculature have been documented [19,20]. These findings highlight the sensitivity of retinal vessels to systemic inflammatory processes.

Based on these insights, we hypothesized that herpes zoster, as an infection associated with local and systemic inflammation, may also affect retinal perfusion measurable by OCTA VD. To date, no studies have systematically examined the effects of herpes zoster on ocular perfusion using OCTA. Our study is the first to employ this non-invasive imaging modality to quantitatively assess microvascular changes in the retina, optic nerve head, and choriocapillaris in patients with trigeminal herpes zoster. By providing detailed VD measurements, we aim to fill a critical knowledge gap regarding early vascular alterations associated with VZV infection in the eye.

## 2. Methods

This study included 48 eyes of 24 patients with newly diagnosed trigeminal HZ who were treated as inpatients in the department of dermatology and 48 eyes of 24 healthy controls matched in terms of gender and age from March 2022 to June 2023. In accordance with national guidelines, the diagnosis was made according to the typical clinical signs: the patients exhibited a moderate to severe unilateral skin rash with typical grouped small blisters on reddened skin in the region of the forehead, temples, eyes, and nose bridge, precisely within the innervation territory of the nervus ophthalmicus (V1) and nervus maxillaris (V2), branches of the trigeminal nerve.

In accordance with existing guidelines [21], the diagnosis of trigeminal herpes zoster was made clinically based on the presence of a characteristic unilateral, dermatomal distribution of painful vesicular lesions, typically preceded by localized neuropathic prodromal pain.

Patients were referred to the department of dermatology by community ophthalmologists, dermatologists, or general practitioners for intravenous antiviral therapy following the acute onset of herpes zoster affecting the V1 and/or V2 dermatomes. Every patient was evaluated clinically by our tertiary university hospital. Hospital-based antiviral treatment was initiated at the time of vesicular eruption, which commonly appeared 24–72 h after the onset of prodromal pain.

The study was approved by the Ethics Committee of the University of Muenster and adhered to the principles of the Declaration of Helsinki (2022-285-f-S). Written informed consent was obtained from all participants after a detailed explanation of the study procedures.

Exclusion criteria included the presence of media opacities affecting imaging quality, vitreoretinal diseases, prior retinal surgery, macular edema, glaucoma, or neurological disorders. A comprehensive ophthalmological assessment was performed for all participants, comprising anterior segment examination, binocular fundus examination, and optical coherence tomography angiography (OCTA). Only patients without signs of intraocular involvement were eligible for inclusion. Individuals presenting with concomitant uveitis, such as anterior chamber inflammation (cells, flare) or retinopathy detected via retinoscopy, were excluded from the study.

Participants provided additional information regarding height, weight, smoking status, and cardiovascular diseases. Patients with diabetes mellitus were excluded due to the known impact of diabetes on ocular perfusion. Individuals with well-controlled arterial hypertension were eligible for inclusion. In cases where patients reported active smoking (*n* = 3), matched control subjects with similar smoking status were included to eliminate smoking as a potential confounding factor. These parameters were collected solely for the purpose of minimizing confounding influences on ocular perfusion and were not part of the primary outcome analysis.

Detailed information on the pre-existing medical conditions of the included patients can be found in Supplementary Table S1.

### 2.1. Optical Coherence Tomography Angiography

OCTA imaging was performed using the AngioVue™ Imaging System (RTVue XR Avanti with AngioVue; Optovue Inc., Fremont, CA, USA). The OCTA data were generated using the split-spectrum amplitude-decorrelation angiography (SSADA) algorithm, as described in previous publications on OCTA technology [22].

The macula was imaged using a 3 × 3 mm OCTA scan; the optic nerve head OCT-angiogram was obtained using the 4.5 × 4.5 mm scan. The device uses a light source centered at 840 nm and operates at a speed of 70,000 A-scans per second. Each 3 × 3 mm scan consisted of 304 × 304 A-scans. The axial and lateral resolution of the system are approximately 5 µm and 15 µm, respectively.

Only OCTA images with a quality index of ≥6 were included in the study, while images with motion artifacts, signal gaps, or poor signal strength were excluded.

The en face OCT angiogram of the superficial capillary plexus (OCTA-SCP) was segmented with an inner boundary set 3 μm below the internal limiting membrane (ILM) and an outer boundary set 15 μm below the inner plexiform layer (IPL). The deep capillary plexus (DCP) en face OCT-A image was segmented with an inner boundary set 15 μm below the IPL and an outer boundary set 70 μm below the IPL. Choriocapillaris (CC) segmentation was defined from 10 μm above to 30 μm below Bruch’s membrane.

After imaging, the VD of the OCTA-SCP, OCTA-DCP, OCTA-CC, the radial peripapillary capillary density (RPC), and the foveal avascular zone (FAZ) area were analyzed using the integrated AngioAnalytics software Version 2018.1.0.43 provided by the device.

All OCTA images were acquired and reviewed by the same three experienced examiners using a standardized protocol to ensure consistency in data collection and analysis. All OCTA examinations were performed during regular outpatient clinic hours, between 8:00 a.m. and 5:00 p.m., to reduce the potential influence of diurnal fluctuations in ocular blood flow [23,24]. The OCTA examinations were performed on the day of admission, ensuring a consistent interval of approximately 1 to 3 days after the clinical onset of herpes zoster across all included patients. This standardized timing enabled uniform data acquisition during the early inflammatory phase of the disease.

Prior to data analysis, an expert reader (EE) reviewed and confirmed the accuracy of the automated segmentation.

### 2.2. Statistics

Data management was conducted using Microsoft Excel 2016, and statistical analyses were performed using IBM SPSS^®^ Statistics 29 for Windows (IBM Corporation, Somers, NY, USA). The normality of the data distribution was assessed using the Shapiro–Wilk test, which indicated a deviation from normality. Consequently, Spearman’s correlation coefficient (rSp) was applied to assess the degree of association between two variables, while the Mann–Whitney U test was utilized for non-normally distributed variables to compare the two groups. Continuous variables were reported as medians with interquartile ranges (25th percentile; 75th percentile). It is important to note that all inferential statistical analyses were exploratory in nature and intended for hypothesis generation rather than confirmatory testing. The significance level was set at *p* < 0.05.

## 3. Results

This prospective study included a total of 48 eyes of 24 patients with newly diagnosed trigeminal herpes zoster (HZ) and 48 eyes of 24 age- and gender-matched healthy controls. There was no significant difference in age between the HZ patient and the healthy control group (*p* = 0.60). The demographic characteristics of patients and healthy controls are shown in Table 1.

### OCTA Findings

**Figure 1:**
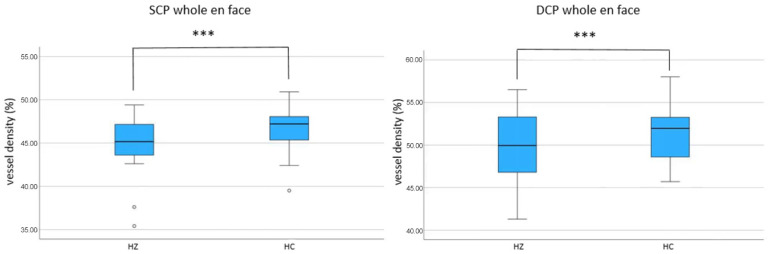
Boxplots illustrating differences in vessel density (VD) between the herpes zoster (HZ) group and the healthy control (HC) group. VD measurements were obtained from the superficial capillary plexus (SCP) and deep capillary plexus (DCP) in the whole en face (WEF) optical coherence tomography angiogram (OCTA). ° indicates statistical outliers; *** indicates statistically significant differences.

The OCTA analyses of the superficial (SCP) and deep capillary plexus (DCP) revealed significant reductions in the VD in all measured areas including the whole en face image (SCP whole en face: patients: 44.85 [42.68; 47.08], healthy controls: 47.35 [45.40; 48.38]; *p* < 0.001; DCP whole en face: patients: 48.85 [44.80; 52.53]; healthy controls: 52.10 [49.40, 53.87]; *p* < 0.001). See Figure 1, which shows boxplots illustrating differences in VD between the HZ group and the healthy control HC group in the SCP.

A significant reduction in the VD was also observed in the optic nerve head (ONH), specifically in the *inside disk* area (patients: 46.20 [42.70, 52.00]; healthy controls: 49.80 [45.63, 52.68]; *p* = 0.047).

Moreover, statistical analysis revealed significant differences between the VD of the patients and controls in the choriocapillaris (CC) whole image (patients: 71.05 [68.03; 72.70]; healthy controls 72.40 [69.30; 74.78]; *p* = 0.024). OCTA data are shown in Table 2.

## 4. Discussion

In this study, we identified a significant reduction in the VD across all analyzed areas of the superficial and deep macular OCT angiogram, as well as in the radial peripapillary capillaries (inside disk) of the ONH in patients with trigeminal HZ compared with healthy controls. This finding expands on the limited existing research on the vascular implications of HZ, which has primarily focused on the effects of HZ on the vasculature of the central nervous system, with no previous studies specifically evaluating ocular microvasculature in HZ patients using OCTA [17,25,26,27,28,29,30,31,32].

**Figure 2:**
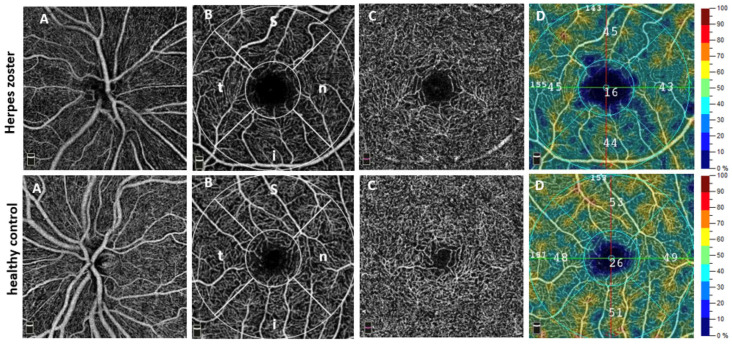
Exemplary optical coherence tomography angiography (OCTA) images of a patient with herpes zoster (HZ) (top row) and a healthy control (bottom row). (**A**): OCTA angiogram of the optic nerve head, depicting the radial peripapillary capillary (RPC) network (4.5 × 4.5 mm). (**B**): En face angiogram of the macula (superficial OCTA angiogram, 3 × 3 mm) with analyzed subregions: parafovea (outer circle) and fovea (inner circle), subdivided into superior (s), inferior (i), nasal (n), and temporal (t) quadrants. (**C**): En face images of the macula (deep OCTA angiogram, 3 × 3 mm). (**D**): Color-coded vessel density (VD) map of the superficial OCTA angiogram. Numbers indicate the VD values measured in the corresponding regions. Note the presence of localized parafoveal ischemic areas (dark blue) visible in the HZ patient image.

Our results now demonstrate significant alterations of the ocular microvasculature in HZ and could provide indications for VZV-induced microangiopathy. Particularly, we found a reduced VD not only in the SCP but also in the DCP, CC, and the ONH. Moreover, OCT images demonstrated localized non-perfusion areas (Figure 2D).

In HZ patients with involvement of the V1 (nervus ophthalmicus) and V2 (nervus maxillaris) branches of the trigeminal nerve, OCTA might therefore help identify ischemic changes that correlate with reduced blood flow, potentially aiding in early intervention. Minassian et al. and Yawn et al. provided evidence for a systemic effect of HZ on blood vessels as they not only showed an increased stroke risk but also an increased risk of myocardial infarction following HZ [29,32].

Potential mechanisms linking HZ to reduced ocular perfusion may involve both direct viral effects and immune-mediated neurovascular injury. Reactivation of VZV from the trigeminal ganglion may lead to a direct viral invasion of the retinal vessels via trans-axonal spread or through hematogenous dissemination during viremia [32,33], resulting in endothelial injury through cytopathic effects.

In parallel, the host’s antiviral immune response triggers a robust inflammatory cascade. Cytokines such as type I interferons (IFN-α/β) play a key role in the innate response to viral infections and have been shown to induce endothelial cell apoptosis and endothelial-to-mesenchymal transition (EndMT), leading to capillary rarefication as described in autoimmune diseases like systemic sclerosis [34,35]. Additional pro-inflammatory mediators, including TNF-α, IL-6, and IFN-γ, may further exacerbate endothelial dysfunction and microvascular inflammation [36,37].

There are only limited data on retinal microvasculature and infections by herpes viruses. Maier et al. investigated differences in retinal VD using OCT angiography in patients with viral anterior uveitis (VAU) caused by the herpes simplex virus (HSV) and VZV and included 88 eyes of 44 patients, divided into equally sized groups [38]. They found reduced VD in the superficial vascular plexus of the macula and peripapillary regions in affected eyes compared with non-affected ones.

Similarly to our study, it highlights reduced VD in HSV- and VZV-affected eyes, reflecting microvascular dysfunction. Both conditions demonstrate that inflammation or direct viral effects may impair retinal microcirculation, potentially contributing to complications like ischemia or neurovascular damage. These findings underscore the diagnostic potential of OCTA for the early detection of perfusion abnormalities, aiding in understanding the vascular impact of viral infections like HZ on ocular health.

Additional studies have shown that chronic systemic inflammatory diseases are associated with retinal microvascular rarefaction detectable by OCTA, supporting the concept that OCTA VD is a sensitive marker for systemic vascular involvement [39]. This aligns with our hypothesis that VZV-induced systemic inflammation and immune-mediated endothelial injury contribute to the observed ocular microvascular impairments.

A key strength of our study is that, to our knowledge, it is the first to demonstrate a significant reduction in ocular perfusion in patients with trigeminal herpes zoster using OCT angiography. By applying a non-invasive, high-resolution imaging technique, we were able to detect microvascular alterations in the retina, choriocapillaris, and optic nerve head at an early stage of the disease. This novel finding not only contributes to our understanding of local ocular involvement in HZ but may also reflect broader systemic microvascular dysfunction induced by varicella-zoster virus reactivation. These findings suggest a potential role for OCTA as a surrogate marker for systemic vascular risk in affected patients. However, further research with larger cohorts and longitudinal study designs will be necessary to confirm this potential and to better evaluate the prognostic value of OCTA for identifying individuals at increased risk of cerebrovascular or cardiovascular complications.

There are certain limitations to our investigation. We only included patients who were hospitalized due to moderate to severe HZ in the department of dermatology. We were not able to recruit outpatients and thus the variability of our HZ group is limited. Likewise, we only included patients with herpes zoster ophthalmicus and no patients with, for example, thoracic herpes. It is possible that herpes in other areas of the body may also lead to reduced blood flow in the eye due to systemic involvement. Unfortunately, we cannot provide longitudinal data on our patients. Future studies with prospective follow-up are warranted to investigate the potential dynamics of ocular perfusion over time following herpes zoster ophthalmicus.

Future research should give priority to such longitudinal studies, especially addressing the question whether alterations of the retinal microvasculature might provide information regarding the risk of future vascular events like stroke or myocardial infarction. However, to address this question, larger cohorts are needed. Comparisons between VD data in the beginning of the disease and after 3–6 months should be part of these investigations.

In conclusion, the ability of OCTA to detect reduced VD in a non-invasive manner provides a window into the microvascular status of HZ patients. Both the stroke/TIA risk demonstrated in previous HZ studies and the reduced ocular perfusion seen in our OCTA study may share same underlying mechanisms of immune-mediated vasculopathy and direct viral effects on vascular structures. OCTA could serve as an early diagnostic tool for assessing the risk of systemic vascular complications in affected individuals, particularly those with trigeminal HZ.

## Figures and Tables

**Table 1:** Demographic and clinical characteristics of the study participants. Values, except for participant counts and gender distribution, are expressed as mean ± standard deviation (SD). Abbreviations: HZ = herpes zoster; n = number; m = male, f = female.

	HZ Group	Control Group	*p*-Value
Subjects, n	24	24	
Eyes	48	48	
Gender f/m	10/14	10/14	
Mean Age (years) ± SD	42.9 ± 16.0	41.6 ± 15.0	0.60

**Table 2:** Vessel density data of the superficial (SCP) and deep capillary plexus (DCP), the optic nerve head (ONH), choriocapillaris (CC), and the foveal avascular zone (FAZ). All values are median (25% quantile; 75% quantile); HZ = Herpes zoster; VD = vessel density; SSI = signal strength index; Bold = statistically significant *p*-values.

OCTA Parameter	HZ Group (*n* = 48 Eyes)	Control Group (*n* = 48 Eyes)	*p*-Value
SCP (VD, %)			
Whole en face	44.85 (42.68; 47.08)	47.35 (45.40; 48.38)	**<0.001**
Fovea	18.90 (16.30; 23.03)	23.10 (19.05; 27.30)	**0.002**
Parafovea	47.50 (45.03; 50.15)	49.65 (47.53; 50.98)	**0.003**
Temporal	46.10(43.80; 48.38)	47.90 (45.83; 50.03)	**0.010**
Superior	49.05 (44.80; 51.38)	50.60 (48.25; 52.35)	**0.023**
Nasal	46.95 (42.93; 49.38)	49.10 (47.60; 50.40)	**0.002**
Inferior	48.45 (46.13; 51.48)	51.40 (49.50; 52.90)	**0.002**
DCP (VD, %)			
Whole en Face	48.85 (44.80; 52.53)	52.10 (49.40; 53.78)	**<0.001**
Fovea	35.05 (32.50; 41.08)	39.10 (34.73; 44.68)	**0.001**
Parafoveal	50.30 (47.55; 54.30)	53.25 (50.98; 55.35)	**0.001**
Temporal	51.55 (48.95; 54.33)	53.60 (51.85; 55.83)	**0.003**
Superior	50.20 (45.53; 54.00)	52.70 (50.90; 55.03)	**0.002**
Nasal	50.25 (48.83; 54.78)	53.55 (51.50; 55.48)	**0.008**
Inferior	49.75 (45.80; 53.98)	53.00 (51.50; 55.90)	**<0.001**
ONH (VD, %)			
Whole en Face	49.25 (47.30; 50.48)	49.55 (47.10; 50.93)	0.548
Inside Disk	46.20 (42.70; 52.00)	49.80 (45.63; 52.68)	**0.047**
Peripapillary	52.45 (50.53; 53.75)	52.05 (50.13; 53.68)	0.739
CC (VD, %)			
Whole image	71.05 (68.03; 72.70)	72.40 (69.30; 74.78)	**0.024**
FAZ (mm^2^)	0.23 (0.14; 0.27)	0.19 (0.12; 0.27)	0.244
SSI Makula	8.00 (7.00; 8.00)	8.00 (8.00; 9.00)	0.527
SSI ONH	8.00 (7.00; 8.00)	8.00 (8.00; 9.00)	0.670

## Data Availability

The original contributions presented in the study are included in the article/Supplementary Material, further inquiries can be directed to the corresponding author.

## Supplementary Materials

The following supporting information can be downloaded at: https://www.mdpi.com/article/10.3390/biomedicines13071630/s1, Table S1: Clinical characteristics of patients diagnosed with herpes zoster.
