# Supplementary material for: Microvascular Density Analysis of Patients with Trigeminal Herpes Zoster—An Optical Coherence Tomography Angiography Study
Source: Biomedicines. 2025 Jul 3;13(7):1630. doi: 10.3390/biomedicines13071630 (PMC12292533; doi:10.3390/biomedicines13071630)
Supplement: Supplementary file 1 [file biomedicines-13-01630-s001.zip › biomedicines-3651686-supplementary.pdf]

Supplementary Table S1. Clinical characteristics of patients diagnosed with herpes zoster.

Patient	Clinical Data				
**Number**	**BMI**	**Arterial hypertension**	**Cardiac diseases**	**Nicotine abuse**	**Other diseases**
1	27.3	No	No	No	migraine
2	35.4	No	No	No	No
3	27.6	Yes	No	Yes, 75 PY	arterial hypertension
4	33	No	No	No	No
5	37.9	No	No	No	atopic dermatitis, chronic venous insufficiency
6	27.8	No	No	No	No
7	24.9	Yes	No	Yes, 20 PY	arterial hypertension, osteoporosis, pancreatic cyst, S/P inguinal hernia,
8	23.9	No	No	No	No
9	21.5	Yes	No	No	arterial hypertension, hypothyreosis
10	22.7	No	No	No	No
11	25.4	No	No	No	No
12	36.6	No	No	No	No
13	26.3	Yes	No	No	hypothyreosis
14	24.1	No	No	No	No
15	29.8	Yes	No	Yes, 20 PY	arterial hypertension
16	28.6	Yes	No	No	No
17	23.1	No	No	No	No
18	19.7	No	No	No	Zenker's diverticulum, Asthma bronchiale
19	29.5	No	No	No	No
20	27.9	No	No	No	No
21	25.5	No	No	No	hypothyreosis
22	26.9	No	No	No	No
23	32	No	No	No	No
24	34.4	No	No	No	Epilepsy
